# Lentiviral vectors as tools to understand central nervous system biology in mammalian model organisms

**DOI:** 10.3389/fnmol.2015.00014

**Published:** 2015-05-18

**Authors:** Louise C. Parr-Brownlie, Clémentine Bosch-Bouju, Lucia Schoderboeck, Rachel J. Sizemore, Wickliffe C. Abraham, Stephanie M. Hughes

**Affiliations:** ^1^Department of Anatomy, Brain Health Research Centre, University of OtagoDunedin, New Zealand; ^2^Brain Research New Zealand Centre of Research ExcellenceDunedin, New Zealand; ^3^NutriNeuro, UMR 1286 INRA, University of BordeauxBordeaux, France; ^4^Department of Biochemistry, Brain Health Research Centre, University of OtagoDunedin, New Zealand; ^5^Department of Psychology, Brain Health Research Centre, University of OtagoDunedin, New Zealand

**Keywords:** lentivirus, temporal and spatial specificity, neuron phenotype, optogenetics, confocal and electron microscopy

## Abstract

Lentiviruses have been extensively used as gene delivery vectors since the mid-1990s. Usually derived from the human immunodeficiency virus genome, they mediate efficient gene transfer to non-dividing cells, including neurons and glia in the adult mammalian brain. In addition, integration of the recombinant lentiviral construct into the host genome provides permanent expression, including the progeny of dividing neural precursors. In this review, we describe targeted vectors with modified envelope glycoproteins and expression of transgenes under the regulation of cell-selective and inducible promoters. This technology has broad utility to address fundamental questions in neuroscience and we outline how this has been used in rodents and primates. Combining viral tract tracing with immunohistochemistry and confocal or electron microscopy, lentiviral vectors provide a tool to selectively label and trace specific neuronal populations at gross or ultrastructural levels. Additionally, new generation optogenetic technologies can be readily utilized to analyze neuronal circuit and gene functions in the mature mammalian brain. Examples of these applications, limitations of current systems and prospects for future developments to enhance neuroscience knowledge will be reviewed. Finally, we will discuss how these vectors may be translated from gene therapy trials into the clinical setting.

## Recombinant Lentiviruses in Neuroscience

Lentiviral biology has been extensively studied since the early 1980s following evidence that human immunodeficiency virus (HIV) was the causative agent of AIDS. Harnessing aspects of this knowledge, gene therapy researchers developed recombinant viral vectors based on HIV (Verma and Somia, 1997; Naldini, 1998), feline, and equine equivalents. Lentivirus is a member of the *Retroviridae* family of viruses, named because reverse transcription of viral RNA genomes to DNA is required before integration into the host genome. Unlike other retroviral genra, such as gamma-retroviruses that are also used in gene therapy, lentiviruses are able to infect both dividing and non-dividing cells by virtue of the entry mechanism through the intact host nuclear envelope (Naldini, 1998; Vodicka, 2001). This characteristic makes it an ideal viral vector for neuroscience, where the majority of cells in the postnatal brain do not divide.

Lentiviral genomes are single-stranded RNA with *gag*, *pol* and *env* genes encoding polyprotein components of the capsid, the enzymes reverse transcriptase, protease and integrase, and envelope glycoproteins, respectively. The viral genome is flanked by long terminal repeats (LTRs), required for genome replication and integration (Naldini, 1998). Lentiviruses have additional accessory genes, but these are dispensable in recombinant vectors. Instead, a recombinant lentiviral vector genome contains LTRs flanking a packaging signal, plus an exogenous promoter used to express a transgene that enables identification of subpopulations of cells, overexpression or knockdown of genes or to target cells with a drug- or light-inducible protein to analyze cell function (Dull et al., 1998). The genome capacity is 8–10 kb for maximal packaging efficiency and viral particles are packaged in human cell lines (usually HEK293 derivatives) by co-transfection of helper plasmids encoding *gag, pol,* and *env* (Dull et al., 1998). In post-mitotic cells, lentiviral vectors integrate at random, whereas integration preferentially occurs into active genes in mitotically active cells (Bartholomae et al., 2011). An alternative and additional safety aspect for post-mitotic cell transduction is the development of integrase deficient lentiviral vectors (Liu et al., 2014). Removal of the integrase from the packaging construct prevents integration, resulting in episomal maintenance of the transgene vector in post-mitotic cells. Recombinant lentiviral vectors appear to offer greater safety over gamma retroviral vectors in which activation of oncogenes has been reported (Hacein-Bey-Abina et al., 2003; Zhou et al., 2010). Although lentiviral vectors have some limitations, mainly in respect to limited spread within the brain parenchyma, this provides an additional advantage in some cases. Permanent integration of lentiviral delivered transgenes into mitotic or post-mitotic cells, similar to episomal maintenance of integrase-deficient lentivirus or AAV in post-mitotic cells, should allow stable transgene expression for the life of the organism or cell, preventing the need for repeated vector administration (Linterman et al., 2011). An important advantage of lentiviral vectors over other vector systems, including adeno-associated virus (AAV), is that inflammatory, and immune responses associated with the vector itself are limited (Abordo-Adesida et al., 2005; Annoni et al., 2007).

This review highlights how lentiviral vectors have been used in neuroscience research. We focus on targeting gene expression to selected neuronal phenotypes, both spatially and temporally, to answer specific biological questions surrounding gene function and the anatomy and physiology of neural circuits in the mature brain.

## Targeting Gene Expression to Structures and Cells

The design of lentiviral vectors has increased in complexity over the last 20 years. As lentiviral vectors have been used as a tool to address more refined neuroscience questions, expectations for increased spatial and temporal accuracy of gene expression have resulted, making it more challenging to design new lentiviral vectors for cutting edge experiments.

Transgene expression can be restricted (i) to certain structures or cell types for refined spatial resolution, (ii) in a constitutive or inducible manner for temporal resolution, and (iii) by activation of the gene product by additional stimuli like light or drugs (post-translational regulation). Below we outline how these restrictions are routinely used to improve knowledge of brain circuitry.

### Spatial Restriction of Gene Expression

**FIGURE 1:**
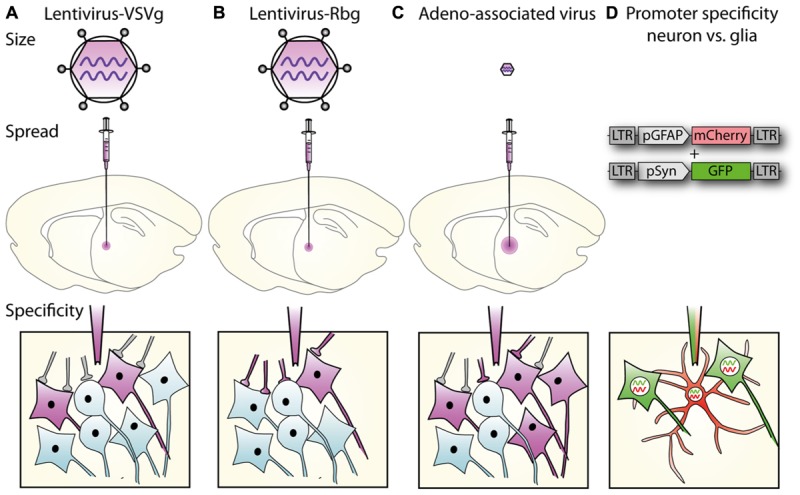
**Lentiviral vector spread, transduction, and expression is mediated by particle size, envelope properties and promoter usage. (A)** Lentiviral vectors pseudotyped with vesicular stomatitis virus glycoprotein (VSVg) mediate local transduction with no retrograde or trans-synaptic viral transport. **(B)** In contrast, lentiviral vectors pseudotyped with a rabies glycoprotein (Rbg) transduce both the soma of local neurons and also afferents to the area. **(C)** AAV, depending on serotype, mediates larger physical spread, and can transduce either local neurons or afferent terminals. **(A–C)** Schematics of relative virus particle size represented in upper panels indicate that lentiviral vectors are approximately 5 × larger than AAVs. **(D)** Transgene expression can be restricted to specific cell types using specific promoters. Using the glial fibrillary acidic protein (GFAP) promoter driving mCherry and synapsin promoter to drive GFP, lentiviral constructs differentially label astrocytes, and neurons, respectively.

In its simplest form, spatial restriction of gene expression is achieved by injecting low volumes and/or titres of the lentiviral vector in the target brain region where its spread will be restricted depending on its tropism and diffusion through the target tissue. Lentiviral tropism is defined by the glycoproteins on the surface of the viral particles that determine which cell surface receptor the virus binds to and thereby the cells or subcellular compartments the virus can enter. Tropism can be modified by pseudotyping, which is the expression of glycoproteins originating from a different virus (Indraccolo et al., 1998; Cronin et al., 2005; Trabalza et al., 2013). The most common pseudotyping method uses the vesicular stomatitis virus glycoprotein (VSVg). VSVg pseudotyped particles have wide tropism as they use low-density lipoprotein receptors to enter cells (Finkelshtein et al., 2013), which are almost ubiquitously expressed on cell membranes (Willnow, 1999), including by both glia and neurons (Jakobsson et al., 2003). By differential pseudotyping, specific populations of neurons within a brain region can be targeted (**Figure 1A**). An example of differential tropism is in the hippocampus, where VSVg pseudotyped vectors mainly transduce cells in the subgranular zone and dentate granule cell layer, while murine leukemia virus glycoprotein (MuLV) pseudotyped vectors more specifically transduce mature granule cells (Watson et al., 2002).

Unlike AAVs, which have a diameter of approximately 20 nm, the larger particle size of lentiviruses (100 nm) limits spread through the extracellular space (Cetin et al., 2006; Lerchner et al., 2014; **Figure 1**), and methods to increase AAV spread, like convection-enhanced delivery or mannitol, are therefore not promising for lentiviral particles. The spread of one microliter of VSVg pseudotyped vector in the brain is restricted to 1–2 mm from the injection site (Desmaris et al., 2001; Linterman et al., 2011), with no retrograde transport. By exchanging the VSVg for a rabies glycoprotein (Rbg; Mazarakis et al., 2001), or a chimera of these two (Kato et al., 2011a,b, 2014; Carpentier et al., 2012; Schoderboeck et al., 2015) retrograde transport is enabled so that after transduction of axonal terminals, viral contents (minus envelope) are transported to the cell body (**Figure 1**).

### Cell-Specific Gene Expression Using Promoters

The promoter controlling gene expression in the lentiviral construct provides a further level of restriction. In addition to ubiquitous promoters, a range of brain cell-type specific promoters are available and well characterized. A list of promoters that are frequently used in neuroscience research is detailed in **Table 1**. Ubiquitous promoters that cause expression of non-native proteins in virtually all transduced cells include elongation factor 1 alpha (EF1α), cytomegalovirus enhancer/promoter element (CMV), β-actin, β-globin chimeric promoter (CAG), and phosphoglycerate kinase promoter (PGK; for a comparison see (Qin et al., 2010) and (Yaguchi et al., 2013). Promoters providing specific expression in all neurons include synapsin 1 (syn; Hioki et al., 2007; Nathanson et al., 2009; van Hooijdonk et al., 2009; Yaguchi et al., 2013) and neuron specific enolase (NSE; Delzor et al., 2012). Neuronal type specific promoters, that have a size compatible with lentiviral vectors are still limited, but include calcium/calmodulin-dependent protein kinase II alpha (CaMKIIα; Dittgen et al., 2004; van Hooijdonk et al., 2009; Seeger-Armbruster et al., 2015), which restricts expression to excitatory glutamatergic neurons and glutamate decarboxylase 67 (GAD67; Delzor et al., 2012) that should restrict expression to inhibitory GABAergic neurons. The parvalbumin promoter targets a subset of GABAergic neurons (Sohal et al., 2009) and ppHcrt targets hypocretin neurons (Zhang et al., 2010). However, because LV-VSVg has strong tropism for excitatory neurons, transgene expression from the GAD67 promoter has also been found in excitatory neurons (Nathanson et al., 2009). The glial fibrillary acid protein (GFAP) promoter is most commonly used to restrict gene expression to astrocytes (Jakobsson et al., 2003), myelin basic protein (MBP) to target oligodendrocytes (McIver et al., 2005), and the nestin (nes) promoter for neural progenitor cells (Beech et al., 2004). The specificity of these promoters is not absolute and can be impaired by viral preparation properties (titre) and promoter properties in certain subregions of the brain. Promoters used in lentiviral vectors typically only comprise minimal promoter sequences, while their endogenous counterparts are often significantly longer and more complex including enhancer and insulator elements. Using a minimal promoter sequence simplifies cloning and helps keep the vector size small, but this also compromises the specificity as parts of the promoter or enhancer and insulator elements will be missing. Integration of vectors close to enhancer or repressor sequences can also impact vector promoter fidelity.

**Table 1:** Commonly used promoters in lentiviruses.

Common abbreviation	Promoter origin	Expressed in	Comments
EF1α	Mammalian elongation factor 1 alpha promoter	Ubiquitous	Endogenous mammalian promoter (Jakobsson et al., 2003).
CMV	Human cytomegalovirus immediate-early enhancer/promoter	Ubiquitous	Methylation-dependent silencing of transgene expression (Brooks et al., 2004)
CAG	CMV coupled with chicken β-actin promoter and first exon and rabbit β-globin splice acceptor.	Ubiquitous	Stable long term expression (Jakobsson et al. (2003), Delzor et al. (2012)).
PGK	Mammalian phosphoglycerate kinase 1 promoter	Ubiquitous	Endogenous mammalian gene Delzor et al. (2012)
MND	Myeloproliferative sarcoma virus enhancer, Negative control region deleted, dl587rev primer-binding site substituted	Ubiquitous	Li et al. (2010), Linterman et al. (2011)
Syn	Mammalian synapsin 1 promoter	Neurons	Dittgen et al. (2004)
GAD67	Mammalian glutamate decarboxylase 67	Inhibitory neurons	Nathanson et al. (2009), Delzor et al. (2012)
CaMKIIα	Mammalian calcium/calmodulin-dependent protein kinase II alpha promoter	Excitatory glutamatergic neurons	Postnatal expression – later in development then synapsin (Dittgen et al., 2004; van Hooijdonk et al., 2009).
NSE	Mammalian neuron-specific enolase promoter	Neurons	Relatively weak expression (Delzor et al., 2012)
MBP	Mammalian myelin basic protein promoter	Oligodendrocytes	McIver et al. (2005, 2010)
GFAP	Mammalian glial fibrilliary acidic protein promoter	Astrocytes	Higher expression in activated astrocytes (Chow et al., 2008).
Nes	Mammalian nestin promoter	Neural progenitor cells	Can also be expressed in activated astrocytes. (Beech et al., 2004; Cheng et al., 2004; Sun et al., 2014).

**FIGURE 2:**
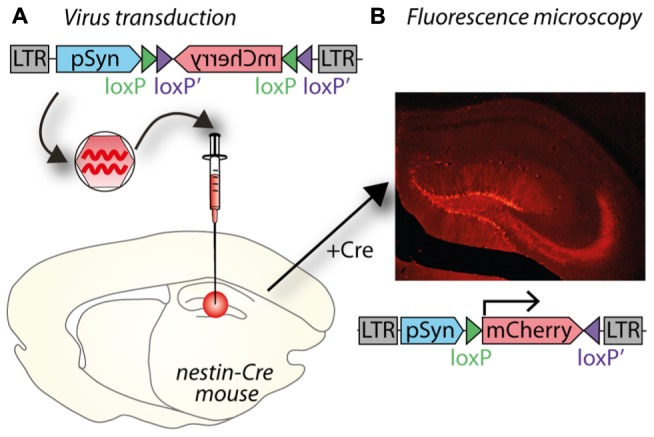
**A potential use of Cre–lox and cell specific promoters to target gene expression both spatially and temporally. (A)** A lentiviral vector (LV- synapsin-double-floxed mCherry) injected in the hippocampal dentate gyrus (DG) of a nestin-cre mouse. **(B)** Cre expressed in nestin-positive neural progenitor cells reverses mCherry and allows expression in new neurons as they differentiate in granule neurons. mCherry expression is therefore restricted to granule neurons born after virus injection.

Tighter cell-type specificity can be achieved by combining lentiviruses with the cre-lox system in transgenic animals. The system involves two components: cre recombinase and a transgene flanked by lox sites (“floxed”), the recognition sites for cre recombinase. The bacterial cre protein uses the lox sites for site-specific recombination in mammalian cells (Sauer and Henderson, 1988). Both components can be supplied by viral vectors or a transgenic animal used to express either cre recombinase, or a floxed transgene; either of which can be controlled by a cell-type specific promoter (**Figure 2**). The lentiviral vector supplies the other component (cre or floxed transgene), which might include a protein under posttranslational regulation (See Posttranslational Control of Gene Expression).

Conditional knockdown of gene expression is another powerful method to analyze protein function in the adult brain. microRNA (miRNA)-based short hairpin knockdown can be combined with cell specific promoters (Nielsen et al., 2009), or used in combination with cre-lox or drug-inducible cre-lox systems for spatial and temporal control of gene knockdown (Stern et al., 2008; Heitz et al., 2014). For example, Heitz et al. (2014) use a lentiviral vector expressing a floxed GFP miRNA to transduce mice expressing GFAP or CaMKII regulated tamoxifen-inducible cre (creERT2).

Further restriction of gene expression can be achieved by miRNA de-targeting. In this system, incorporating miR binding sites into the 3′ UTR of transgene constructs within a viral vector limits expression to those cells that do not express that miR. For example, miR-9 is expressed in all cells in the brain with the exception of microglia. By incorporating miR-9 binding sites into the transgene construct, miR-9 will bind to, and degrade transgene RNAs in all cells except microglia, resulting in selective expression of the transgene in microglia (Akerblom et al., 2013). Similarly, preventing transgene expression in neurons, and enhancing astrocyte expression can be aided by incorporation of the miR-124 binding sequences into the vector (Colin et al., 2009). As further advances in understanding miR biology and cell type specificity emerge, these constructs can be further refined for selective gene expression.

### Temporal Control of Gene Expression

Temporal control of gene expression is achieved using inducible promoters. These promoters are activated by an additional factor that is added to or removed from the system. They are widely used in bacterial expression systems and include promoters controlled by drugs, metal ions, heat shock, or hormones – only some of which are useable in mammalian cells, and even fewer in the CNS where drugs need to cross the blood brain barrier. Most commonly, drug-inducible promoters are used in neuroscience, where the transgene is expressed under the control of a minimal promoter sequence that is only active if bound by a transactivator. The ability of this transactivator to bind the promoter is regulated by co-factors. The most famous and widely used inducible promoter system is the Tet-On/Off system and derivatives thereof. In this system, the transgene is downstream of a minimal promoter under the control of a Tet response element (TRE) based on an *Escherichia coli* operon conferring resistance to the antibiotic tetracycline. For use in mammalian cells, the Tet repressor (Tet-On) is fused to the activating domain of virion protein 16 of herpes simplex virus (VP16) that constitutes the tetracycline-controlled transactivator (tTA). When tTA binds to the TRE, transcription from the minimal promoter is stimulated by tetracycline or its derivative doxycycline (DOX) in a concentration-dependent manner, thus providing a reversible on and off switch (Gossen and Bujard, 1995; Gossen et al., 1995; Pluta et al., 2005). In the Tet-Off system, the tTA binds to the TRE in the absence of tetracycline and activates gene expression; when tetracycline or DOX is administered, gene expression is repressed (Gossen and Bujard, 1992). The Tet-On system was derived by mutation of the tTA leading to the opposite phenotype, where administration of tetracycline induces gene expression (Gossen et al., 1995).

Light-inducible promoters have recently expanded the potential of inducible promoters (Wang et al., 2012). In a system similar to the Tet concept, a photoactivatable transactivator dimerises upon exposure to light of a certain wavelength, permitting it to bind to its response element and thereby induce gene expression. The advantages of this optogenetic system over drug-inducible promoters are the higher spatial and temporal accuracy of induction and reversibility (ms to s resolution), whereas tetracycline (and DOX) is usually delivered systemically and its effects can last from hours to days (Agwuh and MacGowan, 2006).

Some specific research questions require tight spatial and temporal control of gene expression and aim to express the transgene in only a specific subset of very similar cells, which can be optimally addressed by combining several techniques. Studying neurogenesis in the adult mammalian brain requires several techniques to be combined to obtain the necessary specificity. In order to only target newborn granule cells, which are very similar to their developmentally born neighboring granule cells, a cre transgenic mouse can be combined with cell-type specific promoter (**Figure 2**). The transgenic mouse expresses cre recombinase under a nestin promoter only present in neural progenitor cells, which reside in the subgranular and subventricular zones in the hippocampus. To only target newly born cells in the hippocampus, a VSVg-pseudotyped lentivirus with the transgene is injected into the dentate gyrus; in **Figure 2**, the transgene encodes the fluorescent protein mCherry. The transgene is inverted and flanked by two sets of loxP sites to prevent leaky expression in cells without cre recombinase. By placing the transgene under a syn promoter, it will only be expressed in mature neurons born after the injection of the lentivirus. Including a Tet system to induce cre expression only at very specific time points could further enhance temporal resolution (Chen et al., 2009).

### Posttranslational Control of Gene Expression

Posttranslational regulation of transgene expression can be used to control neural activity by using reversibly activatable ion channels or G-protein coupled receptors (GPCR; reviewed in Rogan and Roth, 2011). The expressed channels and GPCRs are responsive to either light or drugs. Below we focus on the utility of light (optogenetic stimulation) to alter cell function or structure because it has the most accurate (shortest) time resolution (ms) and can mimic the time course of real neural activity. However, sophisticated, highly specific and activatable changes in gene expression can occur over longer time frames using drugs (min-days), so are ideally suited for examining biological states such as circadian rhythm, sleep-wake cycle, stress, and the control of feeding. One of the most recent advances is to completely isolate the introduced transgene from the endogenous ligand–GPCR combinations by developing designer receptors exclusively activated by designer drugs (DREADDs; Armbruster et al., 2007). One advantage of DREADDs is that both receptors and ligands are specifically designed to have high affinity to each other, but not with other targets *in vivo*.

#### Light Inducible Control of Neuronal Function – Optogenetics

In the field of neuroscience, a major new application of lentiviral vectors has been the development of optogenetic technology. Optogenetic stimulation (optogenetics) is the result of 30 years of intense research in both gene therapy and light-activated proteins. Since the first article in 2005 describing the use of optogenetics in neuroscience (Boyden et al., 2005), optogenetics has genuinely revolutionized neuroscience research and the number of publications using optogenetics has increased exponentially (Aston-Jones and Deisseroth, 2013). Optogenetics was chosen as the method of the year by the prestigious Nature Methods journal in 2010.

**FIGURE 3:**
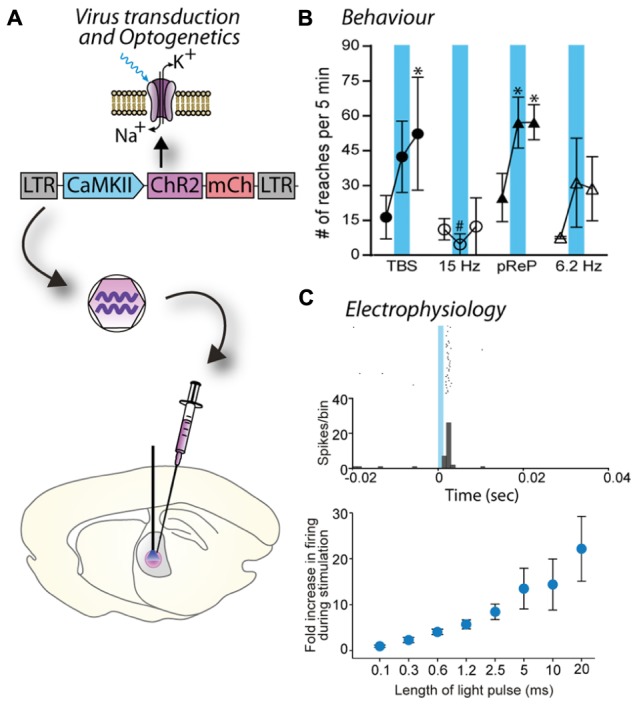
**Blue light stimulation of neurons expressing a channelrhodopsin alters cell activity and behavior. (A)** A lentiviral vector (LV-CaMKII-ChR2-mCherry) was injected into the motor thalamus of rats to transduce glutamatergic neurons. Blue light stimulation of motor thalamus opened channelrhodopsin2 cation channels in the membrane of transduced neurons. **(B)** Effect of blue light stimulation of motor thalamus on movement performance in an acute model of parkinsonism. For each pattern, the number of reaches executed by rats is represented for 5 min before, during and after blue light (473 nm) stimulation. Theta burst stimulation (TBS) and the physiological reaching pattern (pReP) are irregular patterns and significantly increased reaching performance. Conversely, the equivalent tonic patterns (15 and 6.2 Hz, respectively) did not improve reaching. ^∗^*p* < 0.05, versus prestimulation (Tukey’s test). #*p* < 0.05 versus pREP pattern (Tukey’s test). Reproduced with permission from Seeger-Armbruster et al. (2015). **(C)** A similar experiment performed with injection of LV-EF1α-hChR2(H134R)-eYFP into ventrolateral motor thalamus of a rhesus monkey. Upper panel, data show the firing rate (spikes/bin) of one neuron before (-20–0 ms), during (0–1.2 ms), and after (1.2–40 ms) blue light stimulation (1.2 ms pulse). The cell responded with a brief increase of spiking activity just after the pulse of blue light. Bottom panel, the duration of blue light stimulation affected the responses of motor thalamus neurons; longer pulses caused larger increases in firing rate. Reproduced with permission from Galvan et al. (2012).

The principle of optogenetics is to express a light-activated protein in brain cells via a viral vector, such as a lentivirus, and then activate this particular population of brain cells with light of a specific wavelength (**Figure 3A**). The power of optogenetics resides in its spatial, temporal, and neuronal phenotype specificity to control brain cells (Fenno et al., 2011), which was previously lacking despite many attempts to solve it using other neuroscience methods. Precise timing of optogenetic stimulation is achieved by light pulses [using a laser or light emitting diode (LED) as a light source and to deliver the light at a target area of the brain using an optical fiber], at millisecond resolution, which is in accordance with rapid generation and transmission of action potentials at the initial segment or along the axon. Spatial specificity is achieved by locally injecting the viral vector into a specific part of the brain and restricting its spread to only the target nucleus by optimizing the number of injections and volume injected at each site to match the three dimensional shape of the target. In cases when a target site has a neuronal phenotype that differs from surrounding nuclei, spatial specificity is also achieved by using a promoter for that neuronal phenotype. Spatial specificity can be further enhanced by careful positioning of the fiber optic probe for controlled application of light within the target nucleus. Transduction of a specific population of neurons is achieved by pseudotyping viral vectors and promoters as described in Section “Spatial Restriction of Gene Expression” and “Cell-Specific Gene Expression Using Promoters,” respectively. Temporal control of channel activity is dependent on activation of the laser. At first, optogenetics was used to replace electrical stimulation because it achieves the goal of activating specific neuronal circuits to understand how they work with significantly improved accuracy. In contrast, electrical stimulation has the major drawback of activating all excitable tissues in the area, including passing fibers (Bosch et al., 2011). Since 2005, the tools for optogenetics (types and properties of light-activated proteins, promoters, viral vectors, light sources, stimulation, and recording devices) have been rapidly developing enabling scientists to create new paradigms and innovative approaches to address complex scientific questions. In particular, optogenetics can be used to dissect the anatomy and function of neuronal circuits (Atasoy et al., 2012) and induce or restore behaviors (Fanselow and Connors, 2005; Chaudhury et al., 2013; Seeger-Armbruster et al., 2015; **Figures 3B,C**). Moreover, while electrical stimulation has been focused on controlling neuronal activity, optogenetics also allows manipulation of glial cells (astrocytes, microglia), which constitutes an incomparable way to understand the role of glia in the brain (Figueiredo et al., 2011; Li et al., 2013).

More recently, optogenetics has evolved beyond controlling cell excitability (ion channels). Light-activated proteins are able to activate downstream signaling pathways, for example OptoXR, a category of light-activated proteins that are able to activate signaling pathways from G_q_, G_s_, or G_i_ proteins when they are activated by 500 nm-light (Fenno et al., 2011). A photoactivatable version of adenylyl cyclase is also available, which has notably been used to increase levels of glucocorticoids to study stress responses in zebrafish (De Marco et al., 2013). Optogenetics can also work by dimerization or interactions of the light-activated proteins upon light stimulation, which can control an almost infinite variety of signaling pathways and have been used notably to trigger cell migration (Pathak et al., 2013).

Lentiviruses were chosen as the viral vector for the first article describing optogenetics (Boyden et al., 2005). Currently, most studies use either lentiviruses or AAVs as the vector of choice in rodents or monkeys. Despite differences in their transduction, specificity, and spread in the brain (**Figure 1**), the selection of one or other vector is rarely explained in research articles. The main difference between lentivirus and AAV is their ability to diffuse in the brain (Packer et al., 2013). Indeed, AAV transduces a much larger area around the injection site compared to lentivirus, which is usually restricted to the site of injection (**Figure 1**). As a consequence, AAVs are preferred when high expression levels of light-activated proteins are needed in a large area of the brain. AAV also seems to cause higher expression at the cell level by inserting a larger number of copies into each cell (Diester et al., 2011); however, this may not be advantageous. Recent studies suggest that high level of gene transfer with AAV increases the probability of developing aggregates of non-native expressed proteins in cells (Diester et al., 2011) and/or axonal malformation (Miyashita et al., 2013). This is less likely to occur with lentivirus, due to the lower level of transduction. Moreover, high levels of light-activated proteins lead to higher stimulation rates, which can lead to artificial effects and thus bias the conclusions (Hausser, 2014). In contrast, lentiviruses are preferred when spatial specificity is needed, despite lower levels of expression, and when the genetic code for expression of the required protein is large. In mice where transgenic technologies are well established, optogenetics is commonly performed with transgenic mice in combination with AAV, using the cre-lox system. In this configuration, spatial specificity is achieved by the injection of a floxed transgene construct and high level of expression is achieved by using AAV. Overall, the choice of viral vector is complex and depends on the experimental design required to answer the research question and it is particularly important because many optogenetic studies are performed on freely moving animals, addressing complex behavioral questions over weeks or months.

In the few studies performing optogenetics in monkey, lentivirus and AAV have both been used successfully (Han et al., 2009; Diester et al., 2011; Galvan et al., 2012; Lerchner et al., 2014) with the difference that lentivirus leads to a smaller proportion of aggregates and may thus be a better option for long term studies, as is usually the case with monkeys.

In the future, optogenetics may evolve towards more specific stimulation of neuronal populations with fewer side effects, and lentiviral tools may play a key role in this. For basic neuroscience research, one of the ultimate goals of optogenetic stimulation techniques is to mimic activity in a normally functioning neuronal network to understand the neuronal coding responsible for brain function (Seeger-Armbruster et al., 2015). Another perspective is to use optogenetics with very specific patterns of light stimulation (such as theta burst stimulation in the cortex) to promote plasticity and restore normal behavior in a brain with a neurological disorder (Seeger-Armbruster et al., 2015). A potential future extension of optogenetics will be its use in humans. Although lentivirus has a limited transduction capacity compared to AAV, this is less important when combined with optogenetic stimulation because that the area of brain transduced by the vector and stimulated by the fiber-optic probe are of similar size. From this perspective, lentivirus may be preferred for optogenetic stimulation to treat or cure neurological diseases in humans in the future because of its specificity and limited long-term toxicity on brain tissue.

### Exploring Neuronal Circuitry in the Brain

Another field being redefined by lentiviral vectors is neural tracing. Lentivirus provides several advantages with spatially defined expression and combinations of tracers with optogenetic and other functional constructs allowing examination of questions not previously possible using traditional tracing techniques.

Analysis of neuronal circuitry has traditionally relied on neuronal tracers such as biotinylated dextran amine (BDA), phaseolus vulgaris leucoagglutinin (PHAL), wheat-germ agglutinin (WGA), horseradish peroxidase (HRP), cholera toxin, and more recently pseudorabies virus to label neuronal pathways (Callaway, 2008; Huh et al., 2010). Some of these tracers label neurons in a predominantly anterograde [high molecular weight (Mr) BDA, PHAL] or retrograde (low Mr BDA, pseudorabies, cholera toxin, HRP), or bidirectional (WGA) way. While these tracers confer spatial specificity that is dependent on the volume injected, all neurons in the injected region are labeled regardless of the phenotype.

**FIGURE 4:**
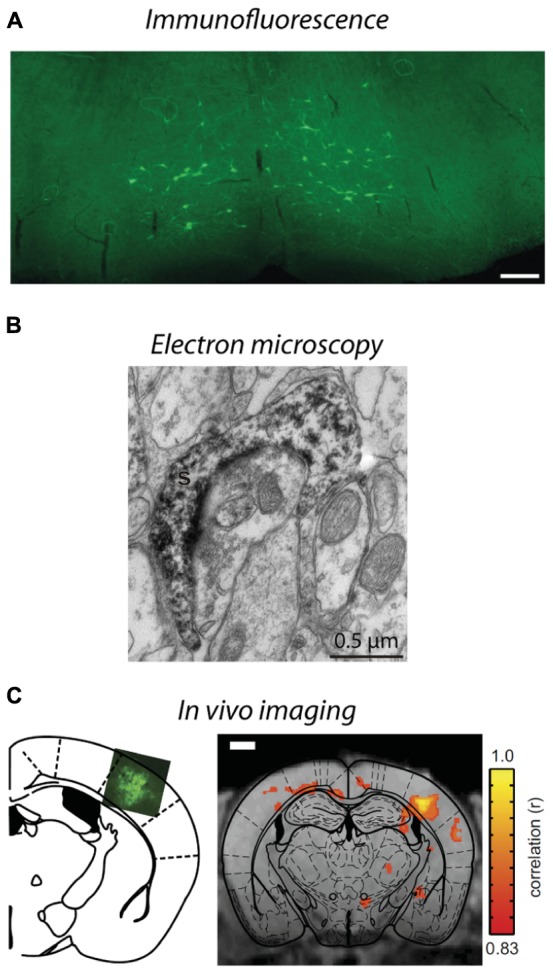
**Visualization of lentivirus transduced neurons in *post-mortem* tissue and *in situ* imaging. (A)** Injection of B19/VSVg-LV-GFP into the lumbar spinal cord resulted in prominent transduction of neurons in the brainstem. Scale bar = 50 μm. Reproduced with permission from Schoderboeck et al. (2015). **(B)** An electron micrograph of a lentivirus transduced spine in the monkey striatum expressing eYFP. eYFP-tagged neuronal elements were made electron dense by using immunoperoxidase labeling with DAB as the chromogen, prior to general processing for electron microscopy. Reproduced with permission from Galvan et al. (2012). **(C)** *In situ* BOLD signals from opto-functional MRI in mice (Desai et al., 2011). Channelrhodopsin-GFP expression following injection of LV-FCK-ChR2- GFP into the somatosensory cortex (left). Voxels with significant increases in BOLD signal (color scale) are shown 1 mm posterior to bregma (right). Reproduced with permission from Desai et al. (2011).

More recently, lentiviral vectors have been combined with microscopy and other biological techniques to investigate the anatomy of neural circuits in many brain areas, including the cerebral cortex, thalamus, basal ganglia, hippocampus, and brainstem (Trono, 2000; Vigna and Naldini, 2000; Duale et al., 2005; Grinevich et al., 2005; Benzekhroufa et al., 2009; Huh et al., 2010; Takada et al., 2013; **Figure 4**). Depending on the lentivirus type and the promoter used in the construct, viral vectors can transduce dendrites, the somata, and terminals of neurons and express proteins at a particular subcellular component, such as in the cell membrane or nucleus (Klein et al., 1998; Gradinaru et al., 2010; Lobbestael et al., 2010; Konermann et al., 2013; Dautan et al., 2014). With careful experimental design, viral vectors can accurately label a particular neuronal phenotype, which affords the possibility of labeling just one distinct pathway in the brain (Tye and Deisseroth, 2012; Dautan et al., 2014; Hasegawa et al., 2014). In addition to spatial specificity, viral vectors allow temporal specificity in neuroanatomy studies, determined by the time of injection and harvesting of tissue (Dull et al., 1998; Takada et al., 2013). In contrast, immunohistochemistry labels all neurons of a phenotype irrespective of the pathways that they belong to and only selected brain slices are stained and analyzed, which means that neurons of interest are only partially visualized (e.g., axon and terminals, but not somata and dendrites, or the converse), unless large numbers of serial sections are analyzed. Viral vectors permit neurons to be transduced in a controlled area of the brain (Huh et al., 2010) because they diffuse smaller distances than traditional neuronal tracers, however, transduction of large populations of neurons can be achieved by injecting a larger volume, injecting multiple times or using a higher titre (Miyoshi et al., 1997; Cai et al., 2013). Furthermore, it is possible to combine viral vectors with conditional systems so that neurons are labeled during a specific period of development (Dull et al., 1998; Wiznerowicz and Trono, 2003). Helper-viruses have also been used in short-term anatomical experiments to specifically label excitatory and inhibitory pathways to better understand whole circuits and to improve cloning capacity (Kumar-Singh, 2008; Liu et al., 2013). For all of these reasons, viral vectors permit unprecedented precise descriptions of pathways and connections for subpopulations of neurons.

As described in Section “Spatial Restriction of Gene Expression,” lentiviral vectors can be pseudotyped with envelopes that modify vector uptake, allowing neuronal tracing studies to be conducted. VSVg-pseudotyped vectors transduce neurons and astrocytes only within the local injection site. Delivery of a cytoplasmic-localized transgene via a VSVg-lentivirus will result in labeling of cell soma within the injection site and fills their projections, allowing tracing of neural target zones. In contrast, Rbg-pseudotyped lentiviral vectors deliver transgenes both locally and via retrograde transport to distal somata with axonal projections localized in the injection zone (Mazarakis et al., 2001; **Figure 1**). The transgene-encoded protein is expressed by transduced neurons – the precise site of expression (e.g., axon, nucleus, cell membrane, histone residues of DNA, etc.) is dependent on where the targeted protein is usually located in the cell. This labeling specificity can be critical because of the complexity of the brain. Here, lentiviral vectors offer improved specificity because most AAV serotypes would label both afferent and efferent neurons in the region (Klaw et al., 2013; **Figure 1**). In addition, the flexibility to have the vectors endocytosed at the terminals or somata of target neurons means that the investigator can select the vector so that injection and uptake is in an area that will not be visualized (Schoderboeck et al., 2015; **Figure 4A**). The advantage for experiments with an anatomical focus is that the physical damage or artifact caused by the injection does not interfere with the interpretation or analysis of images taken at high-resolution, such as electron microscopy. For example, if the somata of neurons will be visualized, injection damage will be minimized by targeting uptake at the terminals of the neurons of interest by using a vector containing a Rbg envelope. Conversely, if the terminals will be visualized, the injection should be targeted at the somata of the neurons of interest by using a VSVg envelope.

Neuroanatomy studies using lentiviral vectors can also be enhanced by combining with other techniques. Generally, the reporter fluorophores expressed by transduced neurons are imaged *post-mortem* using fluorescence or confocal microscopy, and often immunohistochemical staining is conducted to confirm that a subpopulation of neurons has been transduced. However, these techniques can be further exploited to determine which cellular compartments express the proteins (Pastrana, 2011; Konermann et al., 2013), for example using epitope tags (Lobbestael et al., 2010), or to detect structural elements, such as synapses, that cannot be definitively determined using light microscopy (Shu et al., 2011; Galvan et al., 2012). Improved tissue clearing techniques (Hama et al., 2011; Chung et al., 2013; Ke et al., 2013) and microscope optics have enabled visualization of transduced neurons in whole brains, and although this has been done using AAVs so far (Deisseroth and Schnitzer, 2013; Tomer et al., 2014; Yang et al., 2014), lentiviral vectors could be used to provide greater spatial specificity. Electron microscopy is another option that permits visualization of small structural elements and subcellular compartments to investigate circuitry at the ultrastructural level. This can be achieved by immunohistochemically tagging the fluorophore expressed by the viral vector and labeling it with an electron dense chromogen such as intensified diaminobenzidine (DAB, **Figure 4B**), using a genetic tag (miniSOG) that polymerizes DAB when exposed to blue light, or application of immunogold particles during postmortem tissue processing (Grinevich et al., 2005; Sosinsky et al., 2007; Scotto-Lomassese et al., 2011; Shu et al., 2011; Galvan et al., 2012; Dautan et al., 2014; Pollock et al., 2014). By combining lentiviral vectors, optogenetic stimulation, and cutting edge processing techniques for electron microscopy, such as high pressure freezing, the impact of changes in physiology on anatomical circuits can be investigated (Watanabe et al., 2014). Furthermore, because lentiviral vectors produce little to no immune or inflammatory response compared to many other vectors (Blomer et al., 1997), they maintain normal morphology and cellular composition in the area to be investigated, which is critical for studies at the ultrastructural level. This technology could be extended to examine the consequence of activating a G-protein by optogenetic stimulation and imaging how that changes the location of proteins in neurons, thus providing a greater understanding of the function and interactions of proteins at the subcellular level (Pastrana, 2011; Konermann et al., 2013). Thus, viral vectors and lentiviruses in particular, have broad utility to investigate neural circuits at synapse, neuronal, and complete circuit levels.

Importantly, the anatomy of brain circuitry can also be explored *in vivo* by imaging neurons transduced by a viral vector *in situ* using functional magnetic resonance imaging (fMRI) or bioluminescence. Bioluminescence imaging *in vivo* requires a reporter gene to encode a bioluminescent enzyme that generates light, usually in yellow-infrared spectral wavelengths, which are detected by the biosensor without using invasive procedures (Massoud et al., 2008; Shah et al., 2008). Generally, imaging of structures deep within an animal or tissue block is facilitated by having a longer wavelength (Contag and Bachmann, 2002; Zhang et al., 2007). Bioluminescence imaging is limited by its low resolution because light is scattered in body tissues; however, this imaging does provide valuable structural information within an animal (Contag and Bachmann, 2002). Bioluminescence imaging has been used to investigate therapeutic applications of gene expression using AAV, (Contag and Bachmann, 2002), adenovirus (Cho et al., 2005; Massoud et al., 2008), and lentiviral vectors (Deroose et al., 2006). Combining optogenetic stimulation of transduced neurons with fMRI offers a greater understanding of how neural activity alters blood oxygen level dependent (BOLD) signals at stimulated and downstream sites (Adriani et al., 2010; Lee et al., 2010; Desai et al., 2011; **Figure 4C**). In addition, positron emission tomography (PET) has been used to image changes in brain glucose metabolism following optogenetic stimulation to examine functionally connected brain structures (Thanos et al., 2013). Neurons transduced with lentiviral vectors express non-native proteins for at least 6 months (Blomer et al., 1997), enabling changes in structure to be assessed over long periods of time, thus may provide insights to disease processes, for example, changes in structure associated with animal models of neurodegenerative diseases such as Alzheimer’s. An alternative method to trace transduced neurons in more superficial areas of the brain *in situ* is to create a window for imaging transduced neurons via multiphoton microscopy. This usually involves live cell imaging to explore when and how large populations of neurons interact as a network (Shah et al., 2008; Mittmann et al., 2011; Knöpfel, 2012), but could be extended to investigate anatomical studies over time.

In the future, there are many ways that lentiviral vectors could be combined with imaging techniques and exploring these possibilities will greatly improve knowledge of the anatomy of neural circuits. Some applications may include optogenetic stimulation and clearing post-mortem tissue to examine a circuit within large blocks of brain for imaging using confocal microscope. Alternatively optogenetic stimulation combined with high pressure freezing of the tissue and electron microscopy would permit activity-dependent changes in ultrastructural circuitry to be investigated. Enhancing the resolution of *in situ* imaging by fMRI or bioluminescence would greatly improve knowledge of how circuitry changes over time in animal models of neurodegenerative diseases. Future improvements might include development of brain bow-like technology (Weber, 2012) using lentiviral vectors so that multiple pathways and neuronal phenotypes can be investigated in whole brains. The exciting prospect is to explore new combinations of lentiviral gene therapy with optogenetic stimulation, tissue processing, and imaging to address previously unprecedented neuroscience questions about health and disease.

## Current Limitations and Future Prospects

Our understanding of the brain is rapidly expanding. Sophisticated technologies allow us to answer complex questions, understand gene function, and visualize the anatomy of neural circuits. The use of viral vectors is enhancing much of this work. Viral vectors have developed substantially from basic gene addition or knockdown with constitutive promoters to drug and light regulation of gene and protein expression. Further refinements of vectors, promoter regulation and transgenes continue. One recent example illustrating the potential of vectors in neuroscience involves mind-controlled gene regulation (Folcher et al., 2014). Combining EEG recorded brain waves and a computer interface, gene expression and/or optogenetic channel activity can be remotely regulated and changes in behavior can be measured. While this is currently in the proof-of concept phase, such technology has huge implications for the treatment of many brain disorders including epilepsy and Parkinson’s disease.

Clinically, lentiviral vectors have predominantly been used to transduce cells *ex vivo*, which have then been later transplanted as a reservoir for production of useful gene products. To date AAV and lentivirus have been used to treat Parkinson’s disease using standard gene therapy technology and although AAV has been preferred for injection into humans because it does not cause any known pathology, both AAV and lentivirus appear to be safe and well-tolerated by these patients (LeWitt et al., 2011; Palfi et al., 2014). The translatability of lentivirus use is currently limited by its restricted spread in large brains; however, further modification of envelope glycoproteins or injection strategies will alleviate this clinical dilemma in the future.

The frontiers of modern biological science will be expanded by developing new biological tools and refining new technologies by collaborative research teams with skills ranging from molecular biology and viral development to functional neuroanatomy and physiology. Involvement by clinical teams will ensure that these technologies address health-related questions and are readily translated to the clinical setting to improve the health and wellbeing of patients.

## Conflict of Interest Statement

The authors declare that the research was conducted in the absence of any commercial or financial relationships that could be construed as a potential conflict of interest.
